# Heterotopic Ossification in the Middle Finger: A Case Report

**DOI:** 10.1155/2011/323795

**Published:** 2011-09-07

**Authors:** Philip Ian G. Barlaan, Wing-Yuk Ip

**Affiliations:** Division of Hand and Foot Surgery, Department of Orthopaedics and Traumatology, Queen Mary Hospital, Hong Kong University Medical Centre, 102 Pok FuLam Road, Hong Kong

## Abstract

A case of heterotopic ossification developed after traumatic laceration of the middle finger. It is extremely rare for heterotopic ossification to locate in the finger. The diagnosis was accomplished with plain radiographs as well as with MRI and confirmatory study with histopathological microscopic examination which demonstrated nubbin of bone surrounded by fibrovascular connective tissue, and the marrow was predominantly fatty with prominent ectatic vessels and patchy mild lymphocytic infiltrate and gross appearance of partly calcified bony tissue.

## 1. Case Presentation

**Figure 1:**
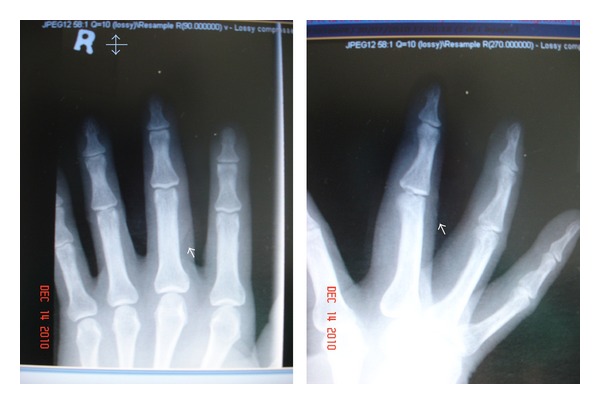
Anteroposterior and lateral views of plain radiographs of the hand. There is no evidence of bony irregularity.

A healthy 57-year-old, male, Chinese with a good past medical health, right hand dominant worked as maintenance worker in a company. While working, a lamp metal bar cover fell on the right middle finger which causes laceration. Upon examination at the Accident and Emergency Department, bleeding wound further had examination under digital block anaesthesia with lacerated wound 3 cm obliquely at the posteroradial aspect of the proximal phalanx and 80% cut of the extensor tendon and no bony involvement. Radiograph findings revealed no bony involvement (Figure 1).

 Emergency surgical exploration at the operating theatre under digital anaesthesia with tourniquet was applied. The operative findings oblique laceration skin wound over posteroradial aspect of the proximal phalanx middle finger right, slight macerated of the skin edge 80% cut of the extensor tendon over the radial aspect (Zone IV), and branch of radial digital nerve was cut. No bony involvement was seen during exploration thus confirmed the plain radiographic findings. The procedures thorough debridement and irrigation, tendon repaired with 2-strands of 4/0 prolene using modified Kessler and 6/0 prolene continuous epitendinous stitch, digital nerve repaired with 4X 8/0 nylon epineurial stitches, and wound closured with 5/0 Nylon.

**Figure 2:**
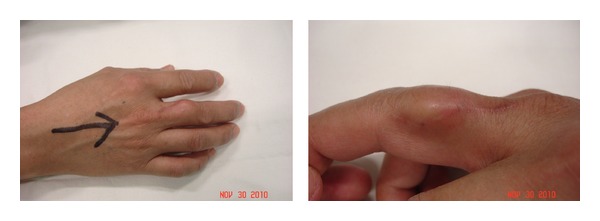
Gross picture four months after the injury. There is a mass at the radio-dorsal aspect of the proximal middle phalanx.

**Figure 3:**
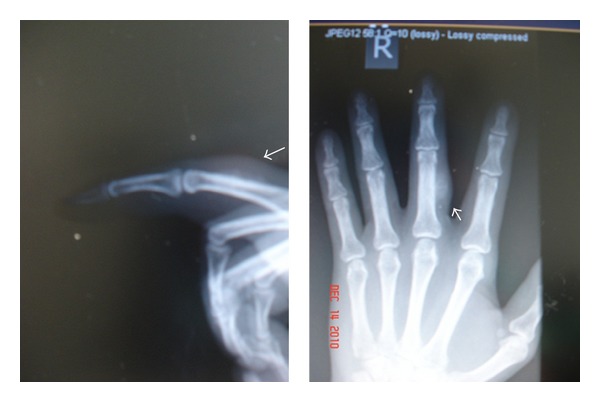
Anteroposterior and lateral views of plain radiographs taken eight weeks after injury. There is a calcified bony tissue.

 Following trauma and surgical procedure, extensive physiotherapy rehabilitation programme and occupational therapy treatment protocol was implemented. After the removal of skin sutures, a palpable mass was noted that begins as a painful over the scar which gradually becomes nontender and gradually increases, as well as firmer to palpation in progressive increase in size at the 19th day after the surgery with continuous extensor programme rehabilitation, and it develops gradual decrease of the range of motion on the metacarpal phalangeal joint as well as the proximal interphalangeal joint even with standard rehabilitation programme; later on, it develop into significant block of flexion and extension (Figure 2). Radiographic imaging taken with calcifications was noted (Figure 3).

**Figure 4:**
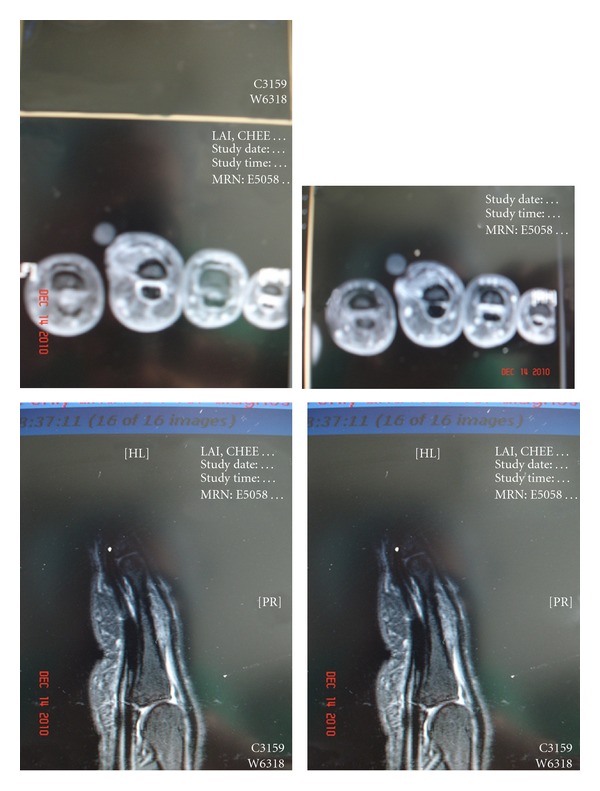
Axial and sagittal FSE T1W, FS T2W, and FS T1W images of the middle finger. There is a heterogenous soft tissue lesion in the radial and extensor aspect of the shaft of proximal right middle finger, measuring 1.0 × 0.7 × 1.8 cm (cross section × long) in size. This lesion is T1W hypointense, T2W heterogeneously hyperintense with some enhancement. This lesion is located underneath the extensor tendon with elevation of the tendon. The extensor tendon is intact and elevated by the mass.

 An MRI + contrast performed after three months showed a finding that there is a heterogenous soft tissue lesion in the radial and extensor aspect of the shaft of the proximal finger, measuring 1 × 0.7 × 1.8 cm (cross section × long) in size. This lesion was T1W hypointense, T2W heterogeneously hyperintense with some enhancement. The MRI finding was a nonspecific local lesion underneath the extensor tendon of the middle finger at site previous injury. The extensor tendon is intact and elevated by the mass (Figure 4).

**Figure 5:**
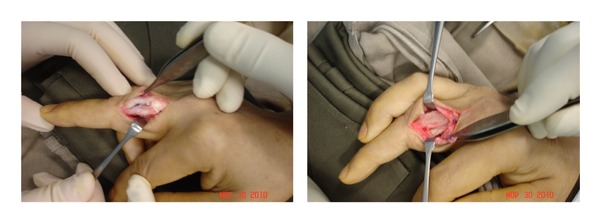
Partly calcified bony tissue up to 18 mm × 10 mm × 8 mm adherent to the proximal phalanx of the middle finger that is located underneath the extensor tendon.

**Figure 6:**
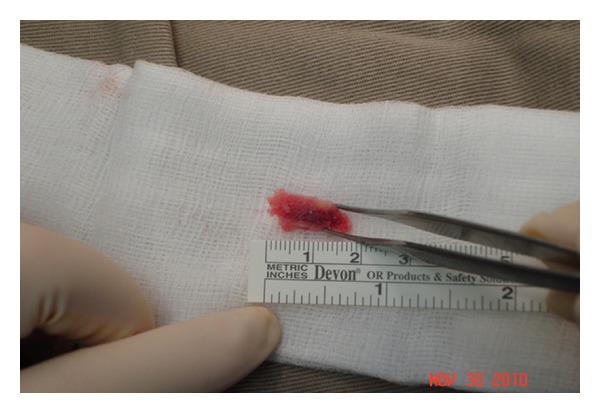
Partly calcified bony tissue up to 18 mm × 10 mm × 8 mm. The microscopic section shows a nubbin of bone surrounded by fibrovascular connective tissue. The marrow is predominantly fatty with prominent ectatic vessels and a patchy mild lymphocytic infiltrate. The decalcification block all embedded.

 This partly calcified bony tissue mass of size up to 18 mm × 10 mm × 8 mm mass was treated by a complete excision and approached the original surgical site at the posteroradial aspect of the proximal middle finger. The extensor tendon healed completely. A bony mass underneath the radial border of the extensor tendon had reached the surface of the proximal phalanx at the radial border (Figure 5). A bony-calcified tissue was removed up to 18 mm × 10 mm × 8 mm in measurement (Figure 6). The underlying proximal phalanx was intact, and there was no evidence of previous fracture. 

 Histological examination section showed a nubbin of bone surrounded by fibrovascular connective tissue. The marrow is predominantly fatty with prominent ectatic vessels and a patchy mild lymphocytic infiltrate. The patient recovered uneventfully after the operation. He regained full range of active and passive movements, and the neurovascular status was intact.

## 2. Discussion

This is a case report with rare location of the HO in a patient with history of isolated trauma of the finger. There has been no literature that reprinted heterotopic ossification in the fingers after isolated traumatic injury. It is uncommon location occurred below the elbows and below the knees. Review of the literature from previous years to the present yielded only Spencer [1] reported a case of HO in finger following head injury. No other reported case was published in the literature. 

 Heterotopic ossification is ectopic bone formation that can be defined as the formation of bone in tissue which normally exhibits no properties of ossification. It is the formation of new bone in tissues which do not normally ossify [2, 3]. When mature, the mass may be densely ossified with a cortex surrounding central trabecular bone [4]. Heterotopic essentially means wrong place, and ossification refers to bone formation. Characterize by progressive abnormal calcified bone formation in soft tissue. It has been defined as the formation of mature lamellar bone in soft tissue. Ectopic bone can develop from immature osteoid in a matter of weeks [5]. Osteoblastic cells responsible for the heterotopic ossification are believed to result from inappropriate differentiation of pluripotent mesenchymal stem cells [6].

 Heterotopic ossification in a finger is uncommon pathological location. The aetiology is unknown and there is no way to predict which patients are more likely to develop. It is a fairly common complication in brain injury and spinal cord injury. It does not occur below the knees nor below the elbows [7]. Multifactorial aetiology with several risk factors was identified which predisposes to its formation. The aetiology of heterotopic ossification can be broadly divided into traumatic, neurological, and genetic, and this case falls into the traumatic group. The most common joints to be affected are the hip, elbow, and shoulder [5]. Heterotopic ossification remains a poorly understood condition with little knowledge of the exact mechanisms involved [5]. In a lower limb arthroplasty, the risk factors which are reported to increase the incidence include male gender, certain pre-existing skeletal affections, and head injury [8] which falls into the traumatic group. 

 Radiography is preferred for initial assessment for virtually all musculoskeletal condition and recommended in all patients with suggested HO to assess underlying bone pathology. A plain radiographs anteroposterior and lateral views can confirm the presence of heterotopic ossification. Plain radiographs will not reveal any abnormality for the initial four to six weeks [9]. It may take up to six weeks for ossification to be evident on radiograph and it is not generally a confirmatory investigation until more than two months after injury [10]. However, radiographs are inexpensive and simple method of assessing the extent of ossification [5]. For early detection and assessment of its maturity, plain radiographs have been largely superseded by three-phase bone scan as early as three weeks after the operation. In our case, with positive calcification in plain radiograph, we did not get any three-phase bone scan uptake study because it is uncommon location to occur and to suspect heterotopic ossification rather MRI was taken for any soft tissue pathology.

 Garland [10] recommends time tables for the surgical removal of heterotopic ossification depending on the aetiology. He advised surgery after six months following traumatic heterotopic ossification, one year following spinal cord injury, and 18 months after head injury [10]. In our case, we removed the heterotopic ossification six months earlier based on the consideration of the range of motion in the finger joints that were limited by the ossification that was present and movement is limited and impaired. It resulted in significant limitation of function owing to joint stiffness. 

 In the literature, treatment may be conservative or operative. The treatment is largely conservative with surgery reserved for patients who do not respond to less invasive measures or who have severe heterotopic ossification [3–11]. Conservative management includes intensive physiotherapy during the maturation phase of the disease process in the attempt to limit the final stiffness it views of the high incidence of recurrence after excision of heterotopic ossification from other joints, a delay in operative treatment is usually advised until the bony mass reaches radiological maturation and biological silence [10], but we believe this is only applicable to bigger joints rather than small joints. Since our case develops limitation of the range of motion of the affected digit due to progressive mass, such a delay will deprive the patient of essential functional capabilities for a long time; it may also increase soft tissue contractures, decrease tendon function, increase risk on neurovascular compromise, further restriction of movement, and less satisfactory restoration of mobility of the joint once the mass of heterotopic ossification has been excised. Late surgical excision of the heterotopic ossified bone to improve the functional outcome and improved the activity of daily living was determined early.

 In our case, we did not postpone surgical excision until six months following traumatic heterotopic ossification or until maturation of the ossification because we believe that finger ectopic bone formation will make difference than in the larger surface area. Our technique for the excision uses a safety-first approach. Adequate exposure is paramount as well as identification of the neurovascular structures. Heterotopic ossification removed adequately from the surrounding soft tissue with sharp surgical dissection. The plane between heterotopic ossification and true cortex was defined by gentle excavation of the ossification with osteotome and curettage, when the true cortex is identified revealed a denser, tougher layer of bone. It is important to aim for complete excision.

 In the immediate postoperative range of movement of the affected metacarpal phalangeal joint and proximal interphalangeal joint considerably improved, and period of continuous passive and active motion in flexion/extension was started to the point of joint resistance and the point of discomfort and it has been shown to be beneficial in improving the eventual range of movement. Active physiotherapy and occupational therapy programme started three days postoperatively. In the patient with HO, careful physiotherapy has been shown to be of benefit [12] because long period of immobilisation is also associated with the formation of heterotopic ossification [13]. Physician should be aware that heterotopic ossification could be possible in the most unexpected location. The surgical excision treatment might not be based on the maturity of the ossified bone but rather functional factor of the affected digit.
